# Purchase decision-making is modulated by vestibular stimulation

**DOI:** 10.3389/fnbeh.2014.00051

**Published:** 2014-02-19

**Authors:** Nora Preuss, Fred W. Mast, Gregor Hasler

**Affiliations:** ^1^Department of Psychology, University of BernBerne, Switzerland; ^2^Center for Cognition, Learning and Memory, University of BernBerne, Switzerland; ^3^University Hospital of Psychiatry, University of BernBerne, Switzerland

**Keywords:** purchase decision-making, insula, neuromodulation, caloric vestibular stimulation, desirability

## Abstract

Purchases are driven by consumers’ product preferences and price considerations. Using caloric vestibular stimulation (CVS), we investigated the role of vestibular-affective circuits in purchase decision-making. CVS is an effective noninvasive brain stimulation method, which activates vestibular and overlapping emotional circuits (e.g., the insular cortex and the anterior cingulate cortex (ACC)). Subjects were exposed to CVS and sham stimulation while they performed two purchase decision-making tasks. In Experiment 1 subjects had to decide whether to purchase or not. CVS significantly reduced probability of buying a product. In Experiment 2 subjects had to rate desirability of the products and willingness to pay (WTP) while they were exposed to CVS and sham stimulation. CVS modulated desirability of the products but not WTP. The results suggest that CVS interfered with emotional circuits and thus attenuated the pleasant and rewarding effect of acquisition, which in turn reduced purchase probability. The present findings contribute to the rapidly growing literature on the neural basis of purchase decision-making.

## Introduction

Purchase decisions are determined by several causes such as availability, preferences and costs. In order to better understand why and when people are buying, recent neuroeconomic studies were able to disentangle different neural components that underlie purchase decision-making (Knutson et al., 2007; Plassmann et al., 2007). Evidence from fMRI studies suggests the involvement of emotional circuits in preferences for and *desirability* of a product (Erk et al., 2002; Paulus and Frank, 2003; McClure et al., 2004; Deppe et al., 2005; Knutson et al., 2007). However, purchase decisions also depend on the actual price of a product and consumers take into account the maximum amount of money they are willing to give up in exchange for a product (*willingness to pay* (WTP)). Behavioral economic theories proposed that acquisition, on the one hand, elicits a feeling of pleasure, but on the other hand it is followed by the immediate *pain of paying* (Prelec and Loewenstein, 1998). However, prices can also be considered as potential gains when making a comparison between different products that can be purchased for the same amount of money (Deaton and Muellbauer, 1980).

The *pain of paying* has been associated with activation in the insular cortex (Knutson et al., 2007). The insular cortex is a phylogenetically old structure. In animal research food stimuli trigger an insular response, which would explain the close relationship of the insular cortex to the gustatory sense (Yaxley et al., 1990; Small, 2010). Several studies have demonstrated that viewing pictures of food activates regions of the insula (Killgore et al., 2003; Simmons et al., 2005; Van der Laan et al., 2012). In humans, the insular cortex developed a more sophisticated function. For example, it plays an important role in anticipation of negative (Simmons et al., 2004; Sarinopoulos et al., 2010; Carlson et al., 2011) and positive events (Lovero et al., 2009). Moreover, neuroimaging studies suggest that the insular cortex is associated with the emotional valuation of objects (Paulus et al., 2003; Knutson et al., 2005; Preuschoff et al., 2008; Rolls et al., 2008; Naqvi and Bechara, 2009). It has strong connections with the orbital network and the middle cingulate cortex (Carmichael and Price, 1995). Furthermore, the orbitofrontal cortex (OFC) is involved in the computation of the final subjective value of an object and is part of a wider valuation network (Wallis and Miller, 2003; Padoa-Schioppa and Assad, 2006; Padoa-Schioppa, 2007; Kennerley et al., 2008; Padoa-Schioppa and Cai, 2011; Walton et al., 2011). The orbito-medial PFC (OMPFC) uses this information to obtain the most desirable and rewarding outcome for the organism (Nauta, 1971). Given the reciprocal connection with OFC, it is by all means possible that input from the insular cortex influences final value computation (Hare et al., 2010).

While previous studies have focused on the role of the prefrontal cortex during purchase decisions and value computation (Camus et al., 2009), results from neuroimaging studies point out an involvement of the insular cortex, the nucleus accumbens (NAcc), the striatum and the anterior cingulate cortex (ACC; Paulus and Frank, 2003; Knutson et al., 2007). These areas have been associated with neural circuits that are associated with reward-related learning and anticipatory reward processing (Paulus et al., 2003; Rogers et al., 2004; Cromwell et al., 2005; Paulus and Stein, 2006). Noninvasive techniques of brain stimulation such as transcranial magnetic stimulation (TMS) cannot be used to stimulate these brain areas as the depth of penetration is limited to 1–2 cm. We examined purchase decision-making during caloric vestibular stimulation (CVS). CVS is commonly used in vestibular diagnostics. However, several studies have demonstrated that CVS also serves as a noninvasive brain stimulation technique to stimulate cortical and subcortical vestibular areas (Bottini et al., 1994, 2001; Deutschlander et al., 2002; Dieterich et al., 2003; Emri et al., 2003; Hegemann et al., 2003; Indovina et al., 2005; Marcelli et al., 2009). Importantly, CVS has been shown to modulate affective control and mood (Dodson, 2004; Levine et al., 2012; Preuss et al., 2014) leading to the conclusion that vestibular areas overlap with emotional circuits. A recent meta-analysis by Lopez et al. (2012) has shown that the insular cortex is the core region activated by means of CVS. Left cold CVS activated more right hemispheric vestibular structures (including the right insular cortex) whereas right cold CVS activated more left hemispheric vestibular structures but less lateralized (Dieterich et al., 2003; Lopez et al., 2012). Miller and Ngo (2007) proposed to use CVS in cognitive neuroscience studies, including studies on decision-making. However, to date, no studies have been carried out to examine the effect of CVS on decision-making, and purchase decision-making in particular. We examined the effect of CVS on purchase behavior, product preferences and WTP.

Taken together, two independent findings motivated us to perform this research. First, Knutson and colleagues demonstrated that right insula activation predicts the decision not to purchase. Second, converging evidence from several neuroimaging studies have suggested that the insular cortex is the core cortical region activated by means of left cold CVS (for overview see Lopez et al., 2012). Therefore, we predicted that right insula activation induced by left cold CVS would reduce the probability of acquisition when compared to sham CVS (Experiment 1). In a second Experiment, subjects rated *desirability* of products (measurement of product preferences) and *WTP* (measurement of economic value computation) while they underwent CVS and sham stimulation. Previous research has shown that activation in the insular cortex is associated with the pain of paying (Knutson et al., 2007) and therefore, we expect that CVS will decrease WTP.

## Materials and methods

### Subjects

Thirty-nine female (mean age = 23.6) subjects took part in the experiment (21 in Experiment 1, 18 in Experiment 2). All subjects were right-handed without any history of neurological or ontological disease. Subjects gave informed consent prior to the experiment and the ethics committee of the University of Bern approved the experimental procedure. Subjects received 30 Swiss Francs (CHF) allowance for participation.

### Task

**Figure 1:**
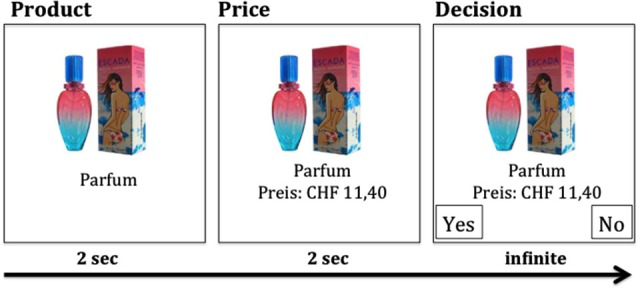
**Each product (total of 120 products) was first presented for 2 s without price information**. After 2 s, the price information was added, followed by the decision option (CHF, Swiss Francs).

In Experiment 1 subjects performed a purchase decision-making task (SHOP task), which was based on the task developed by Knutson et al. (2007). The task consisted of 120 labeled products, which were presented to the subjects by means of a head-mounted display. Products were presented for 2000 ms, followed by presentation of the price for 2000 ms (Figure 1). Then, subjects chose either to purchase the product or not, by pressing the “yes” or “no” button (“f” and “j” keys were used). The side of the response buttons was counterbalanced. The price of the products was 20% of the original market price. Two blocks of 60 randomly selected products were randomly assigned to either the CVS or the sham stimulation condition. Products were equally often assigned to both stimulation conditions. Each product was presented only once per subject. After the experiment, subjects answered questions about desirability (Likert-Scale “1”–“6”; 1 = very low interest, 6 = very high interest), WTP (0-10-20-30-50-70-100% of products’ market price by pressing one out of keys “1”–“7”) and familiarity (Likert-Scale “1”–“6”; 1 = very low familiarity, 6 = very high familiarity) of all products. Both scales were presented from top to bottom. Upon completion of the Experiment 1 out of all trials was determined randomly. If the subject had decided to buy the respective product during that trial, she had to pay for it and received the product by mail.

In Experiment 2 subjects task was to rate the *desirability* of the products and *WTP*. Desirability again ranged from 1 to 6. Subjects used a keyboard for responses and pushed keys from “1”–“6”. WTP ranged from 0 CHF to 100% of market price. The steps were 0-10-20-30-50-70-100% of products’ market price. Subjects pushed keys “1”–“7”. Again, both scales were presented from top to bottom. There was no time limit for responding. Sixty randomly selected products were assigned to either the CVS or the sham stimulation condition. Each product was presented only once per subject.

### Materials

For noninvasive brain stimulation we used an air caloric device (Airmatic II, GN Otometrics, Taastrup, Denmark). Stimulation was performed by inserting a short, flexible plastic tube (length: 2 cm) into the external auditory canal. Subjects were seated in a tiltable chair with a headrest. In order to ensure proper vestibular stimulation, a videonystagmographical device (eVNG, BioMed, Jena, Germany) was used to record the slow phase velocity of nystagmus. Eye movements were recorded for 45 s prior to the experimental block. For visual stimulus presentation we used E-prime 2.0 software and an 800 × 600 resolution head mounted display (i-glasses PC/SVGA PRO 3D, EST, Germany) interfaced with the stimulus computer. Subjects responded by either pressing the “f” or “j” button for “yes” or “no”.

### Caloric vestibular stimulation (CVS)

Subjects were exposed to cold air CVS (20°C) to the left ear. In the sham condition, subjects were exposed to air stimulation at body temperature (37°C) to the left ear, which is physiologally inefficient and does not induce a thermoconvection in the semicircular canal. The order of stimulation conditions was counterbalanced across subjects. Before the experiment began, subjects were tilted forward by 30° from the gravitational vertical. This brings the horizontal semicircular canals into an earth-horizontal plane, which does not give rise to any thermoconvection. After 120 s of CVS, a steady-state thermal gradient in the temporal bone is reached and subjects were pitched backwards by 90° (end position 60° with respect to the gravitational vertical) while CVS continued. This brings the horizontal semicircular canals into an earth-vertical plane in order to maximize thermoconvection (Barnes, 1995; Mast et al., 2006). As soon as the end position was reached, the experimenter started the SHOP task in Experiment 1 or the desirability/WTP rating task in Experiment 2. Vestibular stimulation continued and the subjects kept their fingers placed on the two response keys (“yes” or “no”) in Experiment 1 (index fingers of left and right hand) and on keys “1”–“7” (7 fingers of left and right hand) in Experiment 2. The subjects were seated comfortably in a dentist’s chair. There was a 20 min break between the stimulation conditions. During the break, subjects were encouraged to stand up and relax.

### Statistics

We conducted an item-based and a subject-based statistical analysis for both experiment. We averaged across subjects for the item-based analysis and across products for the subject-based analysis. We performed one-sided *t*-tests for dependent samples to compare means of the CVS and sham condition. Furthermore, we analyzed the data separately for the order of conditions (CVS first followed by sham stimulation an *vice versa*) in order to investigate a possible effect of stimulation order.

In Experiment 1, the dependent variable for the item-based analysis was the purchase probablity of each product depending on stimulation condition. For the subject-analysis, the dependent variable was the percentage of bought products for each subject depending on stimulation condition. Familiarity, desirability and WTP were measured after the actual experiment.

Dependent variables in Experiment 2 were *desirability* and *WTP*. Again, we performed both an item and a subject-based analysis. For the item-based analysis, the dependent variables were the average *desirability* and *WTP* ratings for each product depending on stimulation condition. For the subject-based analysis, the dependent variables were the average *desirability* and *WTP* ratings of each subject depending on stimulation condition. We adjusted the critical α-level using Bonferroni correction, testing both outcomes at an α-level of 0.025.

## Results

### Experiment 1

#### Item-based analysis

**Figure 2:**
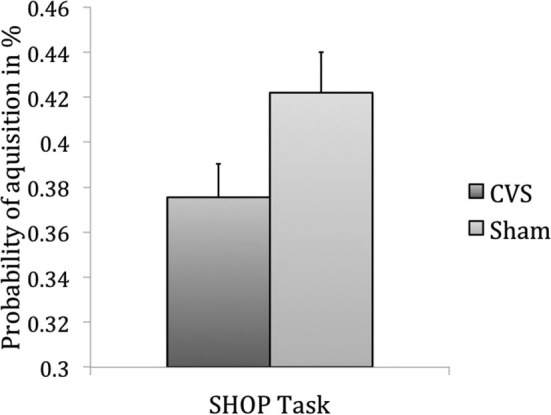
**Results of Experiment 1, item-based analysis: CVS decreased probability of buying a product (*p* = 0.014)**.

In Experiment 1, the dependent variable was the probability of buying a product. Overall probability of purchase was 40% (SEM = 0.01; range 5–76%). Products were bought significantly less often during CVS (mean probability of purchase 37.6%) when compared to sham (mean probability of purchase 42.2%), *T*_(119)_ = −2.24, *p* = 0.014 (Figure 2). Analyzing the products separately for the order of conditions (CVS first followed by sham stimulation and *vice versa*) revealed a decrease in product purchase probability when sham stimulation was first (product purchase probability decreased from 0.44_(sham)_ to 0.33_(CVS)_), *T*_(119)_ = −3.68, *p* < 0.001, whereas there was no significant decrease when CVS was first (decrease from 0.42_(CVS)_ to 0.41_(sham)_), *T*_(119)_ = 0.49, *p* = 0.31.

#### Subject-based analysis

Averaging across products revealed a tendency that subjects bought fewer products during CVS than during sham stimulation, *T*_(20)_ = −1.56, *p* = 0.06. There was no effect of order for the subject-based analysis (both *p*-values > 0.08), but a tendency that the decrease in purchase decision-making was larger for those who started with sham stimulation.

General interest in the products was 2.5 (SEM = 0.031) and products were of medium familiarity (mean = 3.72, SEM = 0.04).

### Experiment 2

#### Item-based analysis

**Figure 3:**
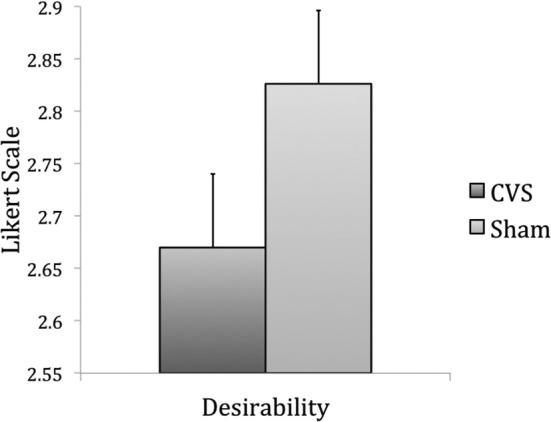
**Results of Experiment 2, item-based analysis: desirability of a product was lower during CVS than during sham stimulation (*p* = 0.01)**.

The same products were used in Experiment 2 (data of one product was missing due to technical problems). Again, the products were randomly assigned to the stimulation condition (CVS or sham). *Desirability* of the products was lower during CVS (mean = 2.67, SEM = 0.07) than during sham stimulation (mean = 2.86, SEM = 0.07), *T*_(118)_ = −2.6, *p* = 0.005 (Figure 3), whereas *WTP* remained unchanged, *T*_(118)_ = −0.2, *p* = 0.42 (CVS: mean = 6.49, SEM = 0.86; Sham: mean = 6.55, SEM = 0.97). Again, the decrease in product *desirability* was higher when sham stimulation was the first condition (from 3.09_(sham)_ to 2.83_(CVS)_), *T*_(118)_ = −3.08, *p* = .002. There were no changes in *desirability* when CVS was the first condition (from 2.65_(sham)_ to 2.57_(CVS)_), *T*_(118)_ = 0.83, *p* = 0.2. There was no effect of order on *WTP* (both *p*-values > 0.04).

The results of Experiment 2 suggest that CVS influences purchase decisions by modulating the *desirability* of products but not *WTP.*

#### Subject-based analysis

There was neither an effect on *desirability* ratings, *T*_(17)_ = −1.04, *p* = 0.15, nor on *WTP* ratings, *T*_(17)_ = −0.45, *p* = 0.33. There was no effect of order in the subject-based analysis on *desirability* and *WTP* ratings (all *p*-values > 0.1).

## Discussion

The results of Experiment 1 show that CVS led to a decreased purchase probability. The probability of buying a product was lower during CVS and there was a tendency that subject bought fewer products when compared to sham, thus confirming the hypothesis. Experiment 2 was designed to disentangle desirability and WPT. D*esirability* of the products but not *WTP* was decreased during CVS.

Noninvasive brain stimulation by means of CVS decreased product purchase probability and purchase decision-making. This was most likely due to altered product *desirability* whereas *WTP* remained unchanged. The results of Experiment 1 are in line with Knutson et al. (2007) who could show that activation of the right insular cortex during the price period predicted subsequent decisions not to purchase. Indeed, all neuroimaging studies using cold left ear CVS revealed activation of the right insular cortex (Lopez et al., 2012). The insular cortex is involved in generating anticipatory signals of a potential negative outcome (Ploghaus et al., 1999). Hence, it is likely that CVS increased the activity in the insular cortex, signaling a possibly “aversive” outcome, and thus decreased probability of product acquisition. To the best of our knowledge, we were the first to investigate the effect of CVS on purchase decision-making.

Experiment 2 was designed to more precisely determine the nature of the processes that led to decreased purchase probability. Knutson et al. (2007) showed that excessive prices elicit increased insular activation leading to the conclusion that the insula is associated with the pain of paying. Interestingly, however, the results show that CVS modulated desirability but not WTP computation.

In the present study, subjects performed a purchase decision-making task with a fixed price (Experiment 1) and a WTP computation (Experiment 2) under CVS and sham stimulation conditions. Stimulation condition modulated the purchase decision outcome but not WTP computation. Interestingly, recent studies have shown that the computation of the economic value of a product is related to frontal areas such as the OFC and the dorsolateral prefrontal cortex (DLPFC; Plassmann et al., 2007). Plassmann et al. (2007) proposed that the economic value of a product is first calculated in the OFC and then passed on to the DLPFC for the final motor decision. Furthermore, prices have no effect on primary taste areas such as the insular cortex (Plassmann et al., 2008). The involvement of frontal areas in the computation of WTP is further supported by findings showing that low frequency repetitive transcranial magnetic stimulation (rTMS) over the DLPFC decreases values assigned to food stimuli (Camus et al., 2009). The results of the present study suggest that brain structures that are activated by CVS are not involved in WTP computation, but they are involved in the emotional evaluation of product preferences and desirability. On an item level, products were judged to be less desirable during CVS when compared to sham. We conclude that the network activated by means of CVS overlaps with emotional circuits, suggesting that the activation of the insular cortex led to an attenuation of the pleasant and rewarding effect of acquisition and therefore decreased purchase probability (Paulus and Frank, 2003; Izuma et al., 2008; Wittmann, 2010). It is noteworthy that the present findings are in line with the valence specific hypothesis. It proposes that the right hemisphere identifies and focuses on discrepancies and is associated with the processing of negative emotions. The left hemisphere in contrast is involved in more goal-oriented behavior, and is associated with positive emotions (Tucker, 1981; Davidson and Fox, 1982; Davidson, 1992; Ramachandran, 1994). These asymmetries can be extended to the insular cortex (Craig, 2005), with the right insula being associated with negative emotions. Based on the results on a subject level, it is therefore possible that left cold CVS brought subjects into a more self-controlled state resulting in a decreased purchase behavior.

Some limitations and further explanations of this study need to be considered. Firstly, it is possible that subjects may have experienced CVS as unpleasant, potentially inducing negative mood. We minimized the occurrence of potentially adverse side effects (e.g., mild nausea) by using air CVS instead of water CVS. Moreover, in a previous study (Preuss et al., 2014) using the identical stimulation method we used the *Positive and Negative Affect Scale* (Crawford and Henry, 2004) and the results showed no increase in negative mood ratings after left ear cold CVS. It is also noteworthy that a negative mood needs not necessarily lead to a decrease in purchase behavior because as subjects may try to regulate their affective state by buying (Rook, 1987; O’Guinn and Faber, 1989; Faber, 1992; Dittmar et al., 2007). Secondly, CVS is an indirect method of brain stimulation, which affects the firing rate of the afferent vestibular nerve that projects to the vestibular nuclei in the brain stem and from there via cortico-thalamic projections to the cortex (Barmack, 2003; Lopez and Blanke, 2011; Lopez et al., 2012). Hence, CVS does not exclusively activate the insular cortex but rather a broad cortical vestibular network with the insula being the core region. Another explanation is that CVS exerted a direct effect on brain structures that are involved in decision-making such as the ACC (Volz et al., 2005). ACC activation did not predict purchase decisions in the SHOP task used by Knutson et al. (2007). However, ACC plays an important role in the processing of conflicting information (Botvinick et al., 2004), and this is for example the case when a very costly product is highly desirable. ACC activation does not necessarily predict how a conflict is solved, but it may facilitate conflict resolution (Knutson et al., 2007) and its activation is associated with risk avoidance (Brown and Braver, 2007). Therefore, it remains possible that CVS had a direct effect on brain structures that are involved in decision-making by having made subjects more risk-aversive. Future imaging studies should further investigate the effect of CVS on ACC and risky decision-making. Furthermore, it would be interesting to examine the influence of CVS on individual differences in purchase behavior by using personality traits as potential moderating variables.

Nevertheless, CVS is a novel method in decision neuroscience that, for the first time, offers a safe possibility to noninvasively stimulate brain areas involved in decision-making that are otherwise not accessible to experimental manipulation. The results suggest that the effect of CVS on purchase decision outcome reflects an overlap of vestibular and affective circuits. In conclusion, CVS attenuated the pleasant and rewarding effect of acquisition by modulating the activity in overlapping emotional and vestibular brain areas.

## Author contributions

Nora Preuss, Gregor Hasler and Fred W. Mast designed the experimental setup. Nora Preuss collected and analyzed the data. Gregor Hasler, Fred W. Mast and Nora Preuss interpreted the results and Nora Preuss wrote the first draft of the article. Fred W. Mast and Gregor Hasler critically revised the article and all authors approved the final version. The authors are accountable for all aspects of the work and ensure that questions related to the accuracy or integrity of any part of the work are appropriately investigated and resolved.

## Conflict of interest statement

The authors declare that the research was conducted in the absence of any commercial or financial relationships that could be construed as a potential conflict of interest.
